# Pulmonary hypertension secondary to pulmonary fibrosis: clinical data, histopathology and molecular insights

**DOI:** 10.1186/s12931-020-01570-2

**Published:** 2020-11-18

**Authors:** Grégoire Ruffenach, Jason Hong, Mylène Vaillancourt, Lejla Medzikovic, Mansoureh Eghbali

**Affiliations:** 1 Division of Molecular Medicine, Department of Anesthesiology and Perioperiative Medicine, David Geffen School of Medicine, University of California, BH-550CHS, Los Angeles, CA 90095-7115 USA; 2 Division of Pulmonary, Critical Care, and Sleep Medicine, David Geffen School of Medicine, University of California Los Angeles, Los Angeles, CA USA; 3 Department of Pathology and Laboratory Medicine, Cedars-Sinai Medical Center, Los Angeles, CA USA

**Keywords:** Pulmonary hypertension, Pulmonary fibrosis, Vascular diseases

## Abstract

Pulmonary hypertension (PH) developing secondarily in pulmonary fibrosis (PF) patients (PF-PH) is a frequent co-morbidity. The high prevalence of PH in PF patients is very concerning since the presence of PH is a strong predictor of mortality in PF patients. Until recently, PH was thought to arise solely from fibrotic destruction of the lung parenchyma, leading to hypoxic vasoconstriction and loss of vascular bed density. Thus, potential cellular and molecular dysregulation of vascular remodeling as a driver of PF-PH has been under-investigated. The recent demonstrations that there is no correlation between the severity of the fibrosis and development of PH, along with the finding that significant vascular histological and molecular differences exist between patients with and without PH have shifted the etiological paradigm of PF-PH. This review aims to provide a comprehensive translational overview of PH in PF patients from clinical diagnosis and outcome to the latest understanding of the histology and molecular pathophysiology of PF-PH.

## Background

Interstitial lung disease, which will be referred to hereafter as pulmonary fibrosis (PF), is an umbrella term that encompasses a wide range of lung diseases which culminate in fibrotic destruction of the lung parenchyma [1]. In the PF patient population, a major determinant of all-cause mortality is the development of pulmonary hypertension (PH) secondary to PF (PF-PH) [2], making PH a potential cornerstone of PF patients care. PH is characterized by sustained elevation of pulmonary pressure as a result of severe pulmonary vascular remodeling and loss of capillary density. In the World Health Organization classification of PH, group 3 includes all PH secondary to lung disease and PF-PH is a subclass (group 3.2) of this group [3, 4].

Our understanding of PH pathogenesis in PF patients primarily relies on data from idiopathic pulmonary fibrosis (IPF) [5]. In the recent years, new pathophysiological concepts emerged that primarily relate to IPF but may also have implications for PH in other forms of PF.

In PH, sustained elevation of pulmonary pressures is driven, at least in part, by sustained inflammation, vascular smooth muscle cell proliferation and angiogenic dysfunction. Different pulmonary vascular remodeling morphologies can be observed in PH [6, 7]. These various morphological changes can both be present in multiple forms of PH or be characteristic of one specific PH subset. Adverse vascular remodeling is observed in all types of vessels throughout the pulmonary vascular tree and most vascular cell-types are involved, with the most prominent being endothelial and smooth muscle cells.

In PF patients, PH was thought for a long time to arise solely from fibrotic destruction of the lung parenchyma, leading to hypoxic vasoconstriction and loss of vascular bed density [8]. Hence, the only relevant approach to treat PH in PF patients was considered to be the restoration of lung parenchymal structure and function, and directly targeting vascular changes was never considered for these patients. The assumed lack of therapeutic relevance in targeting vascular dysfunction in PF-PH patients has led to an under-investigation of its pathophysiology. Recently however, the demonstrations of absent correlation between the severity of fibrosis and development of PH, and of significant histological and molecular differences between PF and PF-PH patients have shifted this paradigm [8–11].

Despite its major role in PF patients’ survival, no clinically approved drugs yet exist to treat PH in PF patients, since all clinical trials thus far yielded results that were inconclusive at best. The lack of approved drugs for PH in PF patients highlights the imperative need for a better understanding of the pathophysiology and molecular mechanisms underlying PH in PF which could lead toward the discovery of effective drugs for this life-threatening condition. This review aims to provide a comprehensive translational overview of PH in PF patients from clinical diagnosis and outcome to the latest understanding of the histology and molecular pathophysiology of the pulmonary vasculature.

## Clinical pathology of PH development in PF patients

PH is characterized by elevated mean pulmonary arterial pressure (mPAP). Until recently, PH was defined as a pathological increase of mPAP ≥ 25 mmHg, but this definition was somewhat arbitrary. In 2018, the 6th World Symposium on Pulmonary Hypertension held in Nice, France [3] updated this definition to take into account new data from mPAP in healthy subjects and the fact that abnormal elevation of mPAP alone is not sufficient to define pulmonary vascular disease, as it may arise from an increase in cardiac output or pulmonary arterial wedge pressure without underlying pre-capillary PH. As such, the new definition includes an increased pulmonary vascular resistance ≥ 3 Wood units in addition to a lower mPAP threshold of > 20 mmHg. Since most research published thus far has used the previous definition of PH, we will still include the previous definition of a mPAP ≥ 25 mmHg for this review. However, readers should keep in mind this new definition as it could change the paradigm of PH in PF patients.

### Prevalence of PH in the PF population

Overall in the different forms of PF, PH prevalence is about 25% in patients referred for lung transplant [12]. However, PF arises from a wide range of causes that influence PH prevalence. PF can be caused by environmental triggers (e.g. asbestos, smoking or occupational exposure), by pre-existing diseases (e.g. Sarcoidosis, connective tissue diseases) or by unknown causes. In Systemic Scleroderma (SSc) patients with PF, Chang et al. [13] have found that 18.2% of SSc patients had PF-PH using echocardiographic estimation of RVSP. Interestingly, in SSc patients with PF-PH there is no correlation between the severity of PF and PH [14]. In Pulmonary Langerhans Cell Histiocytosis (PLCH), which is a smoking-related form of PF, the overall incidence of PH is between 92 and 100% [15, 16].

Recent studies demonstrated that PH is also common in chronic hypersensitivity pneumonitis with a prevalence ranging from 30.8 to 50% [17, 18]. Oliviera et al. [17] demonstrated that contrary to most other forms of PF-PH, PH is correlated to PF severity in chronic hypersensitivity pneumonitis patients, however the relatively small number of patients (50 patients) warrant for larger studies to confirm these results.

In sarcoidosis, the exact prevalence of PF-PH is unknown [19]. In unselected sarcoidosis patients PH has a prevalence of 5–6% at rest [20, 21]. Additionally, in sarcoidosis patients with normal resting pulmonary pressure, the prevalence of PH increases to 43% during exercise [20, 22, 23]. PH in sarcoidosis is however not always concomitant with PF since 40–60% of sarcoidosis patients with PH do not show radiographic signs of PF [24, 25]. In sarcoidosis patients with PF-PH, like in other form of PF-PH, the severity of PH does not correlate with PF severity.[16, 25, 26].

In IPF, PH prevalence in PF is usually investigated in patients listed for lung transplant. These patients are younger and sicker than the average IPF population [27]. Thus, they may not be representative of the general IPF population. Nonetheless, studying this sub-population of IPF patients can still lead to a better understanding of PH development in PF patients.

**Fig. 1:**
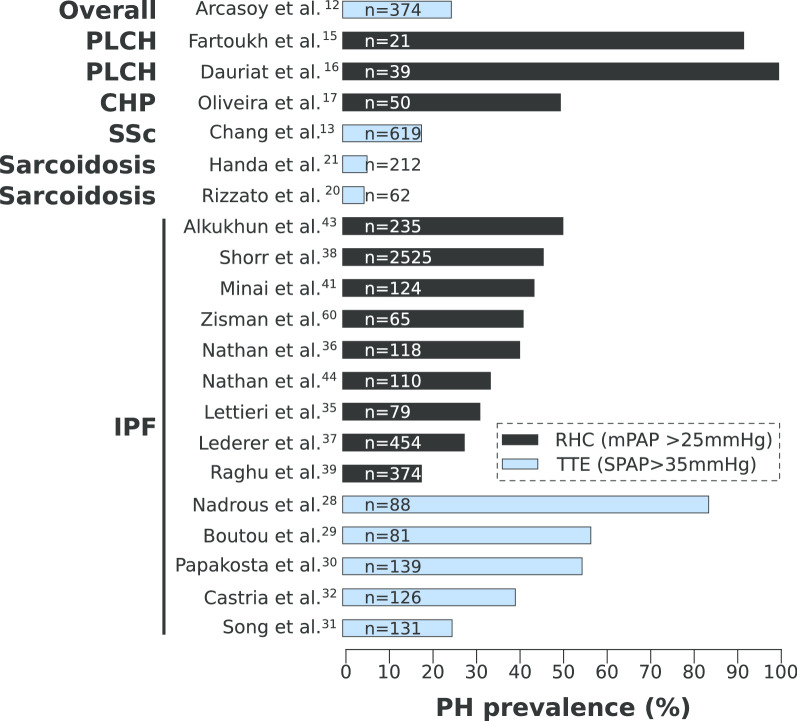
Prevalence of PH in PF patients. Graphical representation of the prevalence of PH found in the PF patient population in multiple studies. Studies in black used right heart catheterization (RHC) with a mean Pulmonary Arterial Pressure (mPAP) above 25 mmHg to define PH. Studies in blue used transthoracic echography (TTE) to define PH with a calculated systolic pulmonary arterial pressure (SPAP) above 35 mmHg except Song et al. which used a SPAP above 40 mmHg to define PH. PLCH (Pulmonary Langerhans Cell Histiocytosis); CHP (Chronic Hypersensitivity Pneumonitis); Ssc (Systemic Scleroderma); IPF (Idiopathic Pulmonary Fibrosis)

In IPF patients listed for lung transplant, studies have found a wide prevalence of PH ranging from 39.7% to 84% using transthoracic echocardiography (TTE, systolic pulmonary arterial pressure > 35 mmHg) to define PH (Fig. 1) [28–32]. TTE is well-known to under- or over-estimate the pulmonary pressure, especially in patients with PF [33, 34] due to the limitations in technique, which will be discussed later in the review. However, TTE limitations may only partially explain the wide range of prevalence observed in these studies. Indeed, in studies using right heart catheterization (RHC, mPAP > 25 mmHg), the gold standard for PH diagnosis, the range of prevalence remains large, from 18.2% to 50.6% (Fig. 1) [35–43]. Thus, other parameters also account for PH prevalence variability between studies. Interestingly, Nathan et al. [44] demonstrated that the prevalence of PH rapidly increases from 38.6% to 86.4% between the initial evaluation for lung transplant and the time of transplant. Therefore, the criteria used to refer patients for lung transplant and the time of data collection may explain variability in PH prevalence.

Among these studies, the largest multicenter study included 2525 IPF patients listed for lung transplant between 1995 and 2004 who underwent RHC [38]. The authors found that PH affected 46.1% of IPF patients, and that supplemental oxygen, pulmonary capillary wedge pressure (PCWP) and forced expiratory volume in 1 s (FEV1) correlated with PH in these patients. The need for more supplemental oxygen in patients could be due to the hypoxemia caused by PH or the disruption of the ventilation/perfusion homeostasis. The correlation between the FEV1 and PH implies a potential functional link between FEV1 and increased mPAP [38]. It remains unclear whether increased mPAP is driving the decrease in FEV1 or vice versa. One could speculate that in the case of eccentric pulmonary vascular remodeling, as observed in PF and PF-PH patients, the increased size of the vascular wall may reduce the lumen of the accompanying airway leading to decreased FEV1. On the other hand, decreased FEV1 signifies airflow limitation which could also participate in elevation of mPAP. Nonetheless, because of the narrow range of FEV1 values (~ 2%) seen in their study, the authors suggest that additional causes to airflow limitation are likely to exist and would explain the raise of mPAP in PF patients [38]. In addition, IPF patients with PH have a significantly increased PCWP compared to PF patients, which suggests a venous component to their condition, although this increase remains subclinical since it does not reach 15 mmHg [38]. By design, these patients do not have left ventricular dysfunction, thus elevated PCWP is likely to be caused by other parameters. One explanation could simply be the accumulation of parenchymal scar tissue leading to reduction of vessel compliance by compressing the pulmonary veins [45]. However, multiple studies also demonstrated vascular remodeling of the veins in PF patients [9, 10, 46], although the molecular mechanism driving this remodeling is not known.

Taken together, while the prevalence of PH encompasses a wide range depending on the patient population and method of evaluation (RHC or TTE), it is evident that PH is largely present in the PF patient population.

### PH: a prognostic factor for survival

The high prevalence of PH in PF patients is very concerning since the impact of PH on survival in IPF patients is well characterized. Lettieri et al. [35] have demonstrated that increases in mPAP (using mPAP > 25 mmHg to define PH), correlate with mortality in IPF patients. In addition, while most studies found that decreased forced vital capacity (FVC) and diffusing capacity of lung for carbon monoxide (DLCO) correlates with mortality in IPF [47–52] whether PH is present or not; Lettieri et al. [20] did not find a significant correlation between mortality and FVC or DLCO. Other studies have investigated whether a mPAP threshold could be used as a prognostic predictor of mortality in IPF patients. In a retrospective study of 101 consecutive IPF patients undergoing RHC at initial evaluation, Kimura et al. [53] found that a mPAP > 20 mmHg was an optimal prognostic predictor. Of note, survival of patients with a mPAP between 20 –25 mmHg and patients with a mPAP > 25 mmHg were similar. In this study, IPF patients with a mPAP > 20 mmHg were significantly younger and had a significant increased pulmo-nary vascular resistance and PCWP. Yagi et al. [54] found similar results, demonstrating that a mPAP > 20 mmHg was a reliable predictor of mortality in IPF patients. In another study, Hamada et al. [55] reported that in IPF patients presenting at initial evaluation for lung transplantation, a mPAP > 17 mmHg was an appropriate cutoff to predict 5-year survival. Thus, even a subclinical increase in mPAP could have severe consequences on survival in IPF patients. Taken together, these data demonstrate that PH is a major predictor of mortality for PF patients. As such, early diagnosis of PH in PF patients could initiate an earlier enlisting for transplant. In addition, it can also facilitate acquisition of clinical data at an earlier stage. These clinical data could help to better understand molecular mechanisms triggering PH development in PF patients paving the way for finding new therapeutic options and improving long-term patient survival.

### PH diagnosis in PF patients

PH is a strong comorbidity in IPF patients, but symptoms of PH are difficult to identify due to lack of specificity [5]. The gold standard for PH diagnosis is by RHC. This procedure is invasive and expensive [56] but relatively safe with low complication rate, particularly when performed at an experienced center [56]. Aside from screening of patients being evaluated for lung transplantation, IPF patients do not routinely undergo RHC, partly because currently there is no approved PH-targeted therapy in this patient population. However, given the significant prognostic implications of PH in the IPF population, PH remains an important co-morbidity to diagnose and various efforts have been made to find reliable, non-invasive methods for PH detection in IPF patients.

#### Pulmonary function testing

In IPF patients, pulmonary function tests are used to evaluate lung function impairment. The FVC and DLCO are the two most common parameters which are assessed in IPF patients. FVC is the quantity of air that can be forcibly exhaled from the lungs after taking the deepest breath possible; and DLCO is the difference of carbon monoxide partial pressure between inspired and expired air. Both parameters are decreased in IPF patients and correlate with disease progression. DLCO can also be decreased in patients with PH without PF. Therefore, in a patient with PF, a decreased DLCO out of proportion to the underlying PF can be a sign of concomitant PH. Until recently, the development of PH in PF patients was predominantly viewed as a result of hypoxic vasoconstriction and destruction of the vascular bed due to the accumulation of scar tissue [57]. However, many clinical reports did not find any correlation between the extent of lung fibrosis and mPAP as measured by high resolution chest computed tomography (HRCT) or FVC in IPF patients [28, 40, 58, 59]. In addition, Nathan et al. [36] reported that DLCO% and FVC% or FVC%/DLCO% ratio were not able to accurately detect PH in IPF patients. Therefore, lung parenchyma destruction and accumulation of scar tissue alone do not explain the development of PH in IPF patients. Interestingly, Zisman et al. [60, 61] have developed a mathematical formula that predicts mPAP from pulmonary function tests using the FVC predicted, DLCO predicted and oxygen saturation. Although, this formula was validated in two distinct small cohorts of IPF patients, the validity of this formula remains to be assessed in a larger cohort of patients. Furthermore, while reduction in DLCO is the most recognized abnormality in pulmonary function tests in patients with PH, this finding is nonspecific given DLCO can also be reduced by other factors such as chronic pulmonary embolism, emphysema, cigarette smoking, pulmonary edema caused by heart failure and anemia. Thus, pulmonary function testing alone has limited predictive value to detect PH in IPF patients [60, 61].

#### Transthoracic echocardiography

Transthoracic echocardiography (TTE) is currently the most commonly used diagnostic test to screen patients for PH as it is a non-invasive cardiovascular imaging tool that can estimate pulmonary hemodynamics. Commonly used indices to detect elevated PA pressure by TTE include peak tricuspid regurgitant velocity, RV outflow tract acceleration time, and early diastolic pulmonary regurgitant velocity [62]. However, in patients with advanced lung disease, TTE has limited accuracy in detecting early stages of PH [12, 63, 64]. In a study of 374 patients awaiting lung transplant, Arcasoy et al. [12] found that using TTE to measure pulmonary vascular pressure in patients with obstructive or interstitial lung diseases was possible in only 44% of the patients. Pulmonary pressure was found to be inaccurate in 52% of this subset, which if taken as the only diagnostic tool, would lead to an overestimation of PH in this population. Fisher et al. [64] also found that in 48% of their cohort pulmonary pressure estimations as measured by TTE were inaccurate, with equally occurring over-and underestimations. This limited accuracy of TTE in estimating pulmonary vascular pressure may be due to challenges in obtaining satisfactory acoustic windows [63]. Therefore, detection of PH in PF patients by TTE can be confirmed by RHC although it may not change the clinical management given there is no approved PH-targeted therapy currently available for this patient population. TTE is also widely used to visualize right ventricular morphology and detect right ventricular dysfunction [65]. Commonly used RV indices to assess for PH by TTE include RV/LV basal diameter ratio to assess for RV dilatation, and tricuspid annular plane systolic excursion (TAPSE), fractional area change, and RV pulsed tissue doppler S wave velocity to evaluate for RV dysfunction [62]. However, RV dysfunction appears in the later stages of PH and is therefore not a suitable readout for early detection of PH.

#### High resolution chest computed tomography

High resolution chest computed tomography (HRCT) scans are routinely performed in PF patients to evaluate the lung parenchyma and have also been used to predict concomitant PH by assessing PA size. One study by Devaraj et al. [63] evaluated CT findings in 30 patients with pulmonary fibrosis with PH. The diagnoses in this group included 16 IPF, 10 nonspecific interstitial pneumonia (NSIP), 3 chronic hypersensitivity pneumonitis, and 1 mixed organizing pneumonia and NSIP. While they found that absolute PA diameter measured by CT did not correlate with mPAP or pulmonary vascular resistance index (PVRi) measured by RHC, they showed that correcting for the size of the ascending aorta (ratio of main PA diameter to ascending aorta diameter) significantly strengthened the correlation to mPAP and PVRi in this group of patients. Furthermore, in a retrospective study of 177 IPF patients undergoing RHC, Yagi et al. [54] found that a ratio of > 0.9 between the diameters of pulmonary artery to ascending aorta measured by HRCT can predict a mPAP > 20 mmHg. A larger and more recent study by Chin et al. [66] included 101 patients with ILD, the majority with usual interstitial pneumonia (the CT and histological pattern seen in IPF) or NSIP. This study found that absolute PA diameter was accurate for detection of PH and correlated with mPAP, contrary to the study by Devaraj et al. [63]. However, main pulmonary artery dilation can also occur in IPF patients without PH [67], thus limiting its use for diagnosis of PH. Perez-Enguix et al. [67] proposed to assess enlargement of the segmental arteries as a sign of PH by HRCT. In this study, they found that three out of four segmental pulmonary arteries should be enlarged to be considered as indicative of PH. Indeed, segmental pulmonary artery enlargement in only one or two lobes could merely be a compensatory mechanism to redirect blood flow away from a fibrotic lobe without affecting the pulmonary pressure [63].

#### Cardiopulmonary exercise test

The cardiopulmonary exercise test is another non-invasive test that may be used for the detection of PH in patients with PF. One single-center retrospective review of 75 PF patients who had undergone cardiopulmonary exercise test and RHC pre-lung transplant found that PF patients with PH had significantly lower end-tidal and mixed-expired carbon dioxide pressure with a distinctive activity pattern for the ratio of these two parameters compared to PF patients without PH [68]. A follow up study by the same group showed that cardiopulmonary exercise test parameters were able to detect differences between levels of severity of PH in patients with PF using the ratio of minute ventilation to rate of carbon dioxide production and end-tidal carbon dioxide pressure [69].

#### Cardiac magnetic resonance imaging

Cardiac magnetic resonance imaging (MRI) is a more advanced non-invasive cardiovascular imaging tool for evaluation of PH available at more specialized centers. In fact, this technology is considered the gold standard in the quantification of right ventricle volumes and function given the limitations of TTE in obtaining optimal acoustic windows in some patients [63, 70]. With respect to PF patients with suspected PH, Chin et al. found diastolic pulmonary arterial area as measured by magnetic resonance imaging was accurate for detection of PH (AUC 0.897) and correlated well with RHC-derived mPAP [66]. The same study also found similar diagnostic accuracy when using pulmonary artery diameter as measured by HRCT pulmonary angiography. Given the limitations of cardiac magnetic resonance imaging which include cost, availability, and need for specialized expertise, TTE may be a more cost-effective cardiovascular imaging test in the initial evaluation of PF patients with suspected PH. However, magnetic resonance imaging may be better for prognostication and risk stratification given its superiority in assessing right ventricular function.

In summary, there is currently no single non-invasive test that is capable of detection and diagnosis of PH with accuracy in PF patients. However, their implementation can lead to the suspicion of PH, which may then be confirmed by invasive RHC.

### Treatment of PH in PF patients

There is currently no approved treatment for PH in PF patients and while PAH specific therapies have been investigated in PF-PH patients, the results were inconclusive at best [71]. A randomized controlled trial [72] investigated the potential benefit of sildenafil, a phosphodiesterase-5 inhibitor, in 180 patients with advanced IPF. Although there was no significant change in 6 min walk distance, sildenafil improved slightly oxygenation and life quality of these advanced IPF patients. Similarly, no benefit was demonstrated during clinical trial investigating endothelin receptor antagonists [73] Bosentan [74] and Ambrisentan [39] in PF-PH patients. Furthermore, a clinical trial testing the guanylate cyclase stimulator Riociguat in PF-PH patients was prematurely terminated due to increased adverse events and risk of death [75, 76]. Finally, in a small clinical trial which investigated the potential of the prostacyclin analogue Treprostinil in PF-PH patients, although there was an improvement in cardiac index and pulmonary vascular resistance [77], 9 out of 15 patients treated with Treprostinil were also receiving phosphodiesterase type 5 inhibitors making any conclusion difficult [78]. Taken together, the failure of these clinical trials strengthen the need for better understanding the molecular mechanism that drives PF-PH for more specific therapeutics.

## Vascular histopathology in PF and PF-PH lung

In PH patients, increased mPAP is driven by pulmonary vascular remodeling and loss of capillary density. Vascular remodeling encompasses multiple vascular morphological changes including vasoconstriction and increased vascular wall thickness due to hypertrophy and/or hyperplasia of the medial and adventitial layers. In PF patients, pulmonary vascular morphological changes occur independently of its cause and adverse vascular remodeling is observed in all types of vessels throughout the pulmonary vascular tree [79]. This section emphasizes similarities and differences in vascular morphological changes found in PF patients with and without PH.

### Similar pulmonary vascular changes in PF and PF-PH lungs

PF and PF-PH patients share similar morphological vascular changes throughout the pulmonary vascular tree as described in this section.

Arteries and arterioles: The elastic pulmonary arteries (> 500 μm diameter) exhibit atheromatous plaque formation [9, 10], similar to what is observed in other forms of PH [79, 80].

All layers of muscular pulmonary arteries (70–500 μm diameter) exhibit concentric and eccentric remodeling. Widespread hyperplasia is present in the intimal layer, which is composed of endothelial cells. The media and adventitia layers are thicker due to hypertrophy and/or hyperplasia of smooth muscle cells and fibroblasts, respectively. In the media, longitudinally oriented smooth muscle cells may be observed, characteristic of hypoxic PH [79]. In addition, these arteries have prominent elastic laminae with pronounced crenation and reduplication of the inner elastic laminae [9–11, 81, 82].

Non-muscularized pulmonary arteries (15–150 μm diameter) exhibit neo-muscularization of the media and luminal narrowing. Anastomoses, a shunt between the pulmonary artery and bronchial systemic artery, are also evident together with hypertrophy of the bronchial arteries [79].

Capillaries: Increased capillary density is evident in normal, non-fibrotic areas of the lung while in the fibrotic area of the lung, vascular regression has been reported by Patel et al.[31]In addition, the distance between capillary and the epithelial basement membrane is increased.

Veins: Finally, pulmonary veins exhibit adventitial thickening, smooth muscle cell hyperplasia and luminal occlusion, as well as intimal fibrosis and thickening of the elastic lamina [9, 10, 81].

### Vascular remodeling specific for the PF-PH lung

All the morphological changes described previously can be found in PF patients with and without PH but they are more pronounced in PF-PH patients. Patel et al. [81] described an increased capillary density in normal lung areas in PF-PH patients. This finding was confirmed by Kim et al. [83] showing that the alveolar capillary density increases concomitantly with the right ventricular systolic pressure. Furthermore, our group demonstrated that in normal areas of the lung, PF patients only exhibited modest vascular thickening compared to healthy controls, while PF-PH patients exhibited significantly increased vascular thickening compared to PF patients [11]. Additionally, pronounced vascular thickness in fibrotic area compared to normal area of the lung was apparent both in PF and PF-PH patients, but PF-PH patients had significantly thicker vascular wall compared to PF patients. These observations confirmed previous reports suggesting that vascular remodeling is mainly limited to fibrotic area in PF patients whereas in PF-PH patients vascular remodeling spreads to the normal area of the lung [9, 83].

To date, only a few studies have investigated morphological changes of the pulmonary vasculature in the normal area of the fibrotic lung, but such studies may provide important insights into the cellular and molecular mechanisms underlying PH development secondary to PF.

## Molecular characteristics leading to vascular remodeling in PF and PF-PH patients

It has been demonstrated that the fibrotic process in PF patients can induce vasoconstriction and impairment of angiogenesis at the molecular level, thus creating a fertile ground for PH development. Recently, robust data showed striking differences at the molecular level between PF and PF-PH patient lungs, thus challenging this deep-rooted paradigm of PH only being driven by fibrotic destruction of the lung parenchyma [46, 84]. However, due to very limited published data on molecular pathophysiology in PF-PH patients, our knowledge and understanding of these molecular differences remains somewhat incomplete. In this section, we will review the key molecular mechanisms common to the fibrotic environment and PH development as well as the recently discovered molecular differences between PF and PF-PH patients.

### The fibrotic process: a fertile ground for PH development

#### Angiogenesis

Proper angiogenic homeostasis is crucial for pulmonary vascular function and is well-known to be impaired in pulmonary arterial hypertension (PAH). Several studies have demonstrated an imbalance between angiogenic and angiostatic factors in the pathogenesis of PF [57, 85]. For example, angiogenic factors such as interleukin-8 or fibroblast growth factor 2 are decreased while angiostatic factors such as pigment epithelium-derived factor, interferon-γ inducible protein 10 or endostatin have been demonstrated to be increased in the lungs of PF patients.

Murray et al. [86] demonstrated that a reduction of vascular endothelial growth factor (VEGF) expression was correlated with poor survival outcome in PF patients, and that apoptotic endothelial cell-derived mediators lead to epithelial cell injury and reduce wound closure. Recently, it was shown that VEGF was decreased in the fibrotic area of IPF patient lungs concomitant with increased endothelial cells apoptosis, which is consistent with the decreased capillary density found in fibrotic regions. In contrast, VEGF is increased in normal, non-fibrotic areas of the lung and is associated with a proliferative phenotype of endothelial cells [87, 88]. These data are in line with the decreased capillary density in fibrotic region but increased capillary density in non-fibrotic region with preserved architecture in PF lungs.

Animal models of PF have also helped in understanding the relationship between fibrosis, VEGF and pulmonary vascular function. Farkas et al. [89] used transforming growth factor β1 (TGFβ1) to induce lung fibrosis in a female rats. In this model, TGFβ1 up-regulation decreased VEGF expression leading to endothelial cell apoptosis and lower vascular density. Up-regulation of VEGF significantly lowered mPAP in these rats, which was concomitant with an increased capillary density and activation of VEGF receptor 2 signaling. Interestingly, VEGF up-regulation also lead to more severe fibrosis. In contrast, in bleomycin-induced mouse model of PF, VEGF overexpression attenuated lung fibrosis [86]. The conflicting results of these studies can be due to differences in the experimental models of PF induced by adenoviral delivery of a mutant TGF-β1 gene in female rats or by bleomycin in female and male mice. Taken together, elucidating the mechanisms underlying dysregulation of angiogenesis could help us understand the transition from a normotensive to hypertensive pulmonary vascular state in PF-PH patients.

#### Bone morphogenetic protein receptor type II

Bone morphogenetic protein receptor type II (BMPR2) is a member of the large TGF-β super-family and regulates growth, differentiation and apoptosis in a diverse number of cell types. BMPR2 plays a major role in PAH wherein its expression is decreased in the lungs regardless of patients carrying a mutation in this gene or not. In PAH, BMPR2 has been shown to play differential roles in pulmonary arterial smooth muscle cell and endothelial cell proliferation, migration and resistance to apoptosis [90]. Furthermore, in PAH patients BMPR2 is downregulated in macrophages, which in turn exhibit increased expression of granulocyte macrophage colony-stimulating factor leading to increased muscularization of the distal pulmonary arteries [91]. Interestingly, Chen et al. [92] found that BMPR2 was also significantly decreased in lung tissue and macrophages of IPF patients, with a greater decrease in IPF-PH group. In this study however, it was not specified if lung tissue was collected from fibrotic or non-fibrotic areas of the lungs. This could be of importance since our group demonstrated that greater histological and molecular differences between PF and PF-PH were found in the non-fibrotic lung areas. Nonetheless, macrophages characterized by decreased BMPR2 signaling and increased expression of the granulocyte macrophage colony-stimulating factor are present in both PF and PH development and could be key players in the vascular changes seen in fibrotic lungs [93].

#### Endothelial to mesenchymal transition

Epithelial to mesenchymal transition (EMT) is the process by which an epithelial cell differentiates into a mesenchymal cell [68]. This process is thought to play a critical role in response to injury [69] and has been implicated in numerous diseases such as PF [94–96]. One subtype ofEMT is the transition of endothelial to mesenchymal cells (EndoMT). EndoMT has been implicated in PAH patients and animal models of PH [97–101]. Ranchoux et al. [97] elegantly described the presence of mesenchymal-like endothelial cells within the intimal layer and vascular lesions of PAH lungs. These cells were characterized by a proliferative and migratory phenotype, accompanied by loss of cell–cell junctions and expression of the well-known EMT transcription factor Twist-1. Interestingly, loss of BMPR2 signaling seemed to be involved in this mesenchymal transition of endothelial cells.

EndoMT has also been demonstrated to participate in the fibrotic process in animal models of PF induced by bleomycin [102], irradiation [103] or endotoxemic injury [104]. A total of 16% of the fibroblast-like cells isolated from bleomycin-induced PF mouse lungs were shown to be from endothelial origin, demonstrating a significant contribution of vascular cell dysregulation on lung fibrosis development. Interestingly, as in PAH, Twist-1 was involved in the mesenchymal phenotype of these endothelial cells, in addition to the other characteristic EMT transcription factor Snail [102]. Choi et al. [103] have shown that TGFβR1/Smad-driven EndoMT was present in the early stage of radiation-induced pulmonary fibrotic process, even before the appearance of fibrotic deposits. EndoMT increased along with fibrosis development, suggesting a causal role for EndoMT in the fibrotic process. Finally, inhibition of EndoMT by vildagliptin prevented pulmonary fibrosis development in mice with PF induced by endotoxemic injury [104]. Together, these data support vascular endothelial dysregulation involvement in the pulmonary fibrotic process, creating a fertile ground for PH development in PF patients.

#### Adenosine

Adenosine is a purine nucleoside, used by cells as a signaling mediator through its binding to four G-protein-coupled receptors. Among these receptors, three are of particular interest: A2AAR and A3AR which are present in the vessel wall [105] and mediate vasodilation [106, 107]; and A2BAR which is expressed by mast cells and macrophages where it participates in the regulation of cytokines expression [105]. In PF, the lung adenosine concentration correlates with fibrosis severity in different animal models of PF [108]. The action of adenosine is mainly mediated by A2BAR in PF and leads to a downstream up-regulation of interleukins 6 and 17 [108]. Interestingly, A2BAR is up-regulated in PF-PH compared to PF patients and its expression correlates with mPAP [107]. On the other hand, adenosine concentration is decreased in the plasma of PAH patients, which participates in vasoconstriction and increases pulmonary arterial pressure [109].

Taken together, these studies demonstrate that PF patients exhibit dysregulation of molecular pathways before the onset of clinical PH. Hence, PF may be viewed as a favorable environment for PH development. However, not all PF patients develop PH, and only a sub-population of PF patients develop PH [28–30, 32–35, 37, 39–41, 43, 44]. Therefore, it is more than likely that other mechanisms aside from fibrotic dysregulation of vascular function participate in PH development in PF patients [9–11, 46, 84].

### A unique molecular signature in the PF-PH lung

**Fig. 2:**
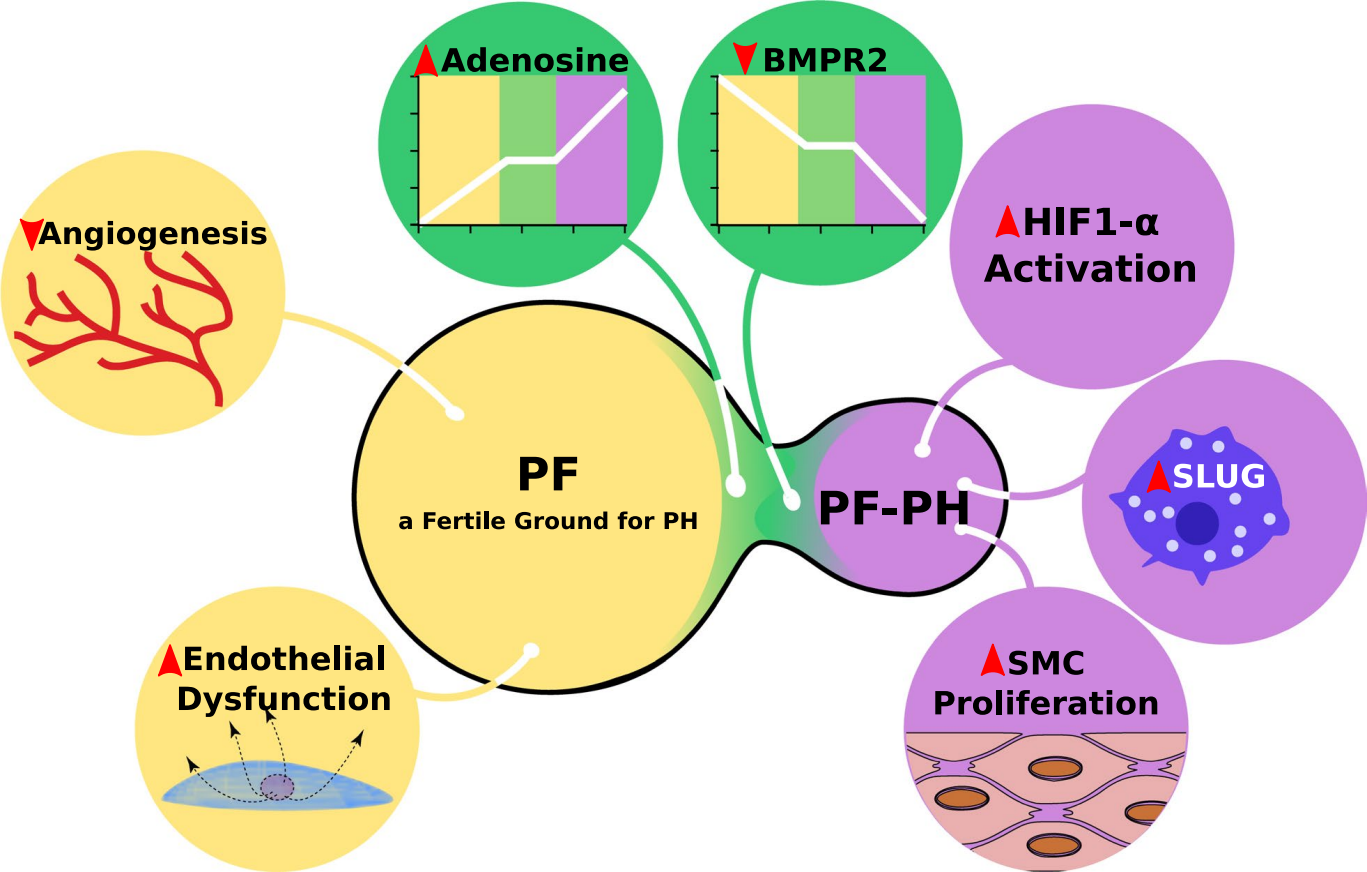
Schematic representation of molecular mechanisms participating in vascular remodeling that promote PH in PF patients. Depicted in yellow, angiogenesis and endothelial dysfunction are known to be dysregulated in PF patients and to participate in PH development, making PF a fertile ground for PH development. Depicted in green, Adenosine and BMPR2 are known to be dysregulated in PF patients and to be further impaired in patients with PF-PH. Depicted in purple, HIF-1a, Slug and pulmonary arterial smooth muscle cells (SMC) proliferation are three hallmarks of patients with PF-PH

Research on molecular differences between PF patients with or without PH remains astonishingly limited. In the past decade, studies in this field slowly started to emerge. This section highlights limited but pioneer work tempting to tease out molecular signature of PF-PH from PF (Fig. 2).

#### Inflammation vs proliferation

Mura et al. [84] are one of the first groups to compare the gene expression of lungs from PF patients with and without PH by microarray. In this elegant study, the authors describe specific gene signatures that differentiate PF and PF-PH patients. The authors found that PF patients without PH mainly had a pro-inflammatory gene signature, while PF patients with severe PH (> 40 mmHg) had a pro-proliferative gene signature. This study demonstrates clear molecular differences between PF patients with and without PH, supporting the hypothesis of specific pathway activation during PH development in PF patients. Interestingly, in patients with mild-to-moderate PH (21–39 mmHg), some patients exhibited a pro-inflammatory gene signature, while others exhibited a pro-proliferative gene signature. This gene signature heterogeneity in the mild-to-moderate group may suggest a transition from pro-inflammatory to pro-proliferative during PH development [54]. On the other hand, Patel et al. [46] performed microarray analysis on distal vasculature from PF and PF-PH, but did not find any significant differences between the two groups. However, their PF-PH patients all belonged to mild-to-moderate PH group, which may still be in line with the study by Mura et al. [84] Another aspect to be considered is the fact that Mura et al. [84] performed their analysis on total lung tissue, while Patel et al. [46] assessed isolated pulmonary distal vasculature. This aspect is of importance, since our group has shown that molecular differences impacting PH development may involve cell types outside the pulmonary vasculature [11]. Therefore, to understand the complex interplay leading to PH development in PF patients, one should consider the lung as a whole rather than focusing only on pulmonary vasculature. In this regard, it could be of interest to use cutting-edge technologies such as single-cell RNA sequencing to better apprehend this complex disease.

Together, these studies support our point of view that PF patients will somehow branch into two different groups: patients who will develop PH and those who will not. Interestingly, Rajkumar et al.[86]have shown that the gene signatures between patients with PF-PH and idiopathic PAH patients are unique. This reinforces our need for a better understanding of PH development in PF-PH patients, as new therapies developed for PAH may not work in this population.

#### Hypoxia-inducible factor 1 alpha

Hypoxia-inducible factor 1 alpha (HIF-1α) is a transcription factor activated in response to hypoxia. When cells sense hypoxia, HIF-1α is translocated to the nucleus and binds to the hypoxia response element to activate the cellular response to hypoxia. In PAH, HIF-1α is activated and participates in the apoptosis-resistant and pro-proliferative phenotype of pulmonary arterial endothelial and smooth muscle cells. In PF patients, HIF-1α is known to be activated throughout the lung parenchyma [110]. However, its activation within the vasculature is more controversial. Garcia-Morales et al. [107] have shown HIF-1α activation in pulmonary smooth muscle cells of PF and PF-PH patients, while Bryant et al. [110] only observed HIF-1α activation in the vasculature of patients with PH. However, in both studies, authors found that the expression of HIF-1α in the vasculature to be significantly increased in IPF-PH patients compared to IPF. Although these two studies report a different pattern of HIF-1α expression in IPF patients pulmonary vasculature, they both clearly demonstrate the presence of HIF-1α within the vasculature of PF-PH patients. HIF-1α activation may be caused by a decrease in the local concentration of oxygen in PF-PH compared to PF. This notion is supported by the study of Shorr et al. [38], which demonstrated that the need of oxygen in PF patients awaiting lung transplant independently correlated with the presence of PH. Another cause for HIF-1α activation in PF-PH may also be dysregulation in oxygen sensing, since it was demonstrated that HIF-1α is activated in normoxic conditions in PAH [111].

However, these hypotheses have yet to be investigated specifically in the context of PH secondary to PF. It is also interesting to note that mice with a specific knock-out of HIF-1α in pulmonary arterial endothelial cells when exposed to bleomycin develop PF similarly to wild-type mice, but do not exhibit elevated pulmonary pressure [110], further supporting a specific role of HIF-1α in the development of PH.

#### Slug/PIP axis

Our group has recently highlighted molecular differences between PF patients with and without PH [11] in addition to the histological characteristics described previously [80]. We found a significant increase of the transcription factor Slug within the lung macrophages of PF-PH patients compared to patients with PF alone [11]. Moreover, we described a remarkable new role for Slug up-regulation within the macrophages, which induces expression of the extra-cellular matrix prolactin-induced protein (PIP). PIP, in turn, promotes proliferation of endothelial and vascular smooth muscle cells. In addition, we demonstrated that bleomycin did not recapitulate the histological features of human in combined PF-PH patients. In the bleomycin-induced PF animal model, vascular remodeling is mainly restricted to fibrotic area of the lung similar to what is observed in PF patients. However, in PF-PH patients vascular remodeling is also evident in non-fibrotic preserved area of the lung. To overcome this translational hurdle in animal models, we developed a new pre-clinical animal model of PF-PH based on the serial administration of bleomycin and monocrotaline. Monocrotaline, an endothelial toxin extracted from Crotalaria spectabilis seeds, has been used for more than four decades to induce PH in rats. Using this combined model, we were able to recapitulate human histological features specific to PF-PH lungs. Furthermore, we demonstrated that this new rat model reproduced the Slug/PIP activation in macrophages as seen in the lungs of patients with PF-PH, in contrast to the model of bleomycin alone. More importantly, we found inhibition of lung Slug was able to prevent PH development in PF rats without affecting lung fibrosis. To the best of our knowledge, this is the first time that a potential treatment was tested in this unique pre-clinical model of PF-PH which could be effective for prevention of PH in PF patients [11].

## Conclusion

In the past decades, most research has focused on the clinical aspects of increased pulmonary pressure for PF patients. Until recently, cellular and molecular data were interpreted as the consequence of fibrotic environment on the pulmonary vasculature. Studies that deciphered differences between PF patients with and without PH remained extremely limited. With a growing body of evidence on specific molecular mechanisms driving PH development in PF patients, the paradigm is slowly shifting from a “passive state”, where PH development was only due to hypoxic vasoconstriction and loss of vascular bed density, to an “active process” where specific molecular and cellular players are involved (Fig. 2). Our understanding of these mechanisms is still in its early days, and an extensive research to deepen our knowledge is crucial to find specific drugs for this life-threatening disease.

## Data Availability

Not applicable.

## Abbreviations

PF: Pulmonary fibrosis
PF-PH: Pulmonary hypertension secondary to pulmonary fibrosis
PH: Pulmonary hypertension
IPF: Idiopathic pulmonary fibrosis
mPAP: Mean pulmonary arterial pressure
TTE: Trans-thoracic echocardiography
RHC: Right Heart catheterization
PCWP: Pulmonary capillary wedge pressure
FVC: Forced vital capacity
FEV1: Forced expiratory volume in 1 s
DLCO: Diffusing capacity of lung for carbon monoxide
RV: Right ventricle
HRCT: High resolution chest computed tomography
MRI: Magnetic resonance imaging
AUC: Area under the curve
VEGF: Vascular endothelial growth factor
TGFβ1: Transforming growth factor β1
BMPR2: Bone morphogenetic protein receptor type II
EMT: Epithelial to mesenchymal transition
endoMT: Endothelial to mesenchymal transition
A2AAR: Adenosine A2A receptor
A3AR: Adenosine A3A receptor
A2BAR: Adenosine A2B receptor
HIF-1α: Hypoxia-inducible factor 1 alpha
PIP: Prolactin inducible protein
